# Influence of Intrinsic Myocardial Conduction on Paced QRS Morphology During Cardiac Resynchronization Therapy Follow up

**Published:** 2008-08-01

**Authors:** Rajendra Deshmukh, K Latchumanadhas, Ajit S Mullasari, Ulhas M Pandurangi

**Affiliations:** Institute of Cardiovascular Diseases, Madras Medical Mission, 4A, Dr. J.J.Nagar, Mogappair, Chennai - 600 037. India

**Keywords:** Cardiac resynchronization therapy, wide QRS, hyperkalemia, myocardial disease

## Abstract

We report two cases of patients of cardiac resynchronization therapy (CRT) whose ECGs, during follow up, showed different paced QRS morphology as compared to those of immediate post-device implantation. Parameters of leads, including sensitivity and capture thresholds, were unchanged. There was no lead dislodgement confirmed on fluoroscopy. The ECGs obtained in device off mode showed different intrinsic QRS morphology as compared to those of pre-implant morphology. These changes were attributable to electrolyte imbalance in one patient and progressive intraventricular conduction defect in the other. These cases demonstrate that intrinsic myocardial conduction pattern influences paced QRS morphology. Irreversible change in paced QRS morphology may indicate poor prognosis.

## Introduction

Change in the paced QRS morphology in patients with CRT devices can be due to loss of capture, dislodgement of ventricular pacing leads, change in the pacing timing of ventricular leads (V-V delay) as well as electrolyte and acid base imbalance without significant change in threshold [1,2]. Influence of change in intrinsic myocardial conduction pattern on paced QRS morphology has not been studied well so far.

Here we present two case reports in which change in paced QRS morphology was attributable exclusively to change in the intrinsic myocardial conduction pattern without change in lead parameters or position. In one case the change in intramyocardial conduction pattern was due to electrolyte imbalance and in another it was due to progression of myocardial disease.

## Case 1

A 66 years old diabetic male with ischaemic cardiomyopathy had undergone CRT for symptomatic congestive heart failure despite optimal medical management. His baseline ECG (1a) showed sinus rhythm, complete RBBB with QRS duration of 130ms. The post-implant ECG (1b) showed sinus rhythm and paced QRS duration of 100 ms with North West QRS axis. Left ventricle was set to be paced 40 ms earlier than RV, to achieve optimal synchronization based on echocardiographic parameters.

The patient was doing well for 18 months following CRT, then presented to us with altered sensorium of 6 hours duration. There was no history of dyspnea, angina or palpitation. His ECG (2a) showed atrial paced and biventricular paced rhythm. However the paced QRS morphology was significantly different as compared to previous post-implant ECGs. The QRS duration (210 ms) was wider by 110 ms. Interrogation of the device revealed no significant changes in lead parameters and ventricular capture thresholds. Fluoroscopy revealed no lead dislodgement.  ECG (2b) obtained when the device was temporarily switched off showed wider QRS complexes (150 ms) as compared to pre-implant ECG with prolonged PR interval (260 ms) and left axis deviation.

His biochemical parameters revealed hyperglycemia (random blood sugar 400mg/dl) and hyperkalemia (serum potassium 6.5 mEq/L). Ketones were present in blood and urine. Renal parameters were mildly deranged (urea 50 mg/dl, creatinine 1.6 mg/dl). He was treated for metabolic encephalopathy with diabetic ketoacidosis. Over a period of 36 hours when biochemical and electrolyte parameters returned to normal, patient became asymptomatic, with clear sensorium. The morphology of paced QRS complex on surface ECG as well as that of intrinsic QRS became identical to that of immediate post-implant ECG.

## Case 2

A 38 years lady with idiopathic dilated cardiomyopathy underwent CRT for refractory heart failure despite optimal medical management. Pre-implant ECG showed (3a) - sinus rhythm, prolonged PR interval (220ms), complete LBBB, QRS duration of 120ms and left axis. Post-implant ECG (3b), after optimizing her A-V & V-V delays showed sinus rhythm, QRS duration of 100 ms and left axis. Left ventricle was set to be paced 40 ms earlier than RV. She was doing well for 15 months after which she started becoming breathless. Breathlessness gradually worsened over a period of one month and she presented to us with complaints of orthopnea and reduced urine output. On admission patient was in pulmonary oedema and her ECG (4a) showed atrial sensed, biventricular paced rhythm. However the paced QRS morphology was significantly different and wider as compared to previous post-implant ECGs. The QRS complexes were of RBBB morphology with superior axis. The QRS duration (180 ms) was wider by 60 milliseconds. Lead(s) dislodgement or capture failure was suspected.

However device interrogation and fluoroscopy ruled out lead and device related issues. The ECG (4b) obtained when the device was temporarily switched off showed wider QRS complexes (168 ms) with prolonged PR interval (200 ms) and atrial enlargement as compared to pre-implant ECG. Echocardiography revealed severe biventricular dysfunction with increase in size of chambers as compared to one-year follow up. She had mildly elevated renal parameters (urea 47 mg/dl, creatinine 1.4 mg/dl) with normal electrolytes. She was stabilized with diuretics and additional inotropic support. After 10 days, at the time of discharge, the QRS morphology (on and off pacing) continued to be wider. Patient ultimately succumbed to progressive pump failure 2 months later.

## Discussion

In addition to mechanical synchrony, electrical synchrony is also achieved in majority of patients with CRT [3,4]. Electrical synchrony manifests on ECG as reduction in the width of QRS [5,6]. The changes in the paced QRS morphology can be due to loss of capture, lead dislodgement, electrolyte imbalance and drugs like amiodarone [1,2].  The changes in the morphology of QRS can also be due to progression of intrinsic disease involving myocardium and conduction system [7]. Hyperkalemia in CHF is usually a consequence of associated renal dysfunction or drug therapy (angiotensin converting enzyme inhibitors, angiotensin receptor blockers, potassium sparing diuretics etc.) or both. Hyperkalemia causes changes in the paced QRS morphology by the following mechanisms. An increase in the concentration of extra cellular potassium lowers the resting membrane potential, leading to a reduction of the upstroke velocity, amplitude, and duration of the action potential. This effect has been recorded in atrial, ventricular, and Purkinje fibers and is manifest on the ECG by changes in the amplitude and duration of the P wave and the QRS complex [8]. A mild to moderate hyperkalemia causes increase in myocardial excitability but further increase leads to impaired myocardial responsiveness to pacing stimulus [9]. It may produce various degrees of exit blocks [10,11]. In our first patient renal impairment and angiotensin converting enzyme inhibitor were considered to be the culprits responsible for hyperkalemia. Measures initiated to normalize potassium levels, promptly restored the paced QRS morphology as well as intrinsic QRS morphology.

Progression of myocardial disease per se has been shown to progressively widen the QRS complex in idiopathic dilated cardiomyopathy [12,13]. Increasing size of cardiac chambers, and concomitant involvement of conduction system by the disease process has been implicated for the change in QRS morphology [14,15]. In our second case the change in intrinsic QRS morphology was due to progression of underlying disease. The fulminant course leading to death in this patient suggests that change in the paced QRS morphology in the absence of known causes is a sign of poor prognosis.

To the best of our knowledge there has been no published case reports describing hyperkalemia and progressive myocardial disease as reasons for changes in the paced QRS morphology in CRT patients.

## Conclusion

Irreversible change in the paced QRS morphology during CRT follow up can be due to gradual progression of underlying cardiomyopathy. This phenomenon is indicative of poor prognosis. Reversible changes in the paced QRS morphology carry a benign prognosis.  These changes may be due to underlying electrolyte abnormality like hyperkalemia.

## Figures and Tables

**Figure 1a:**
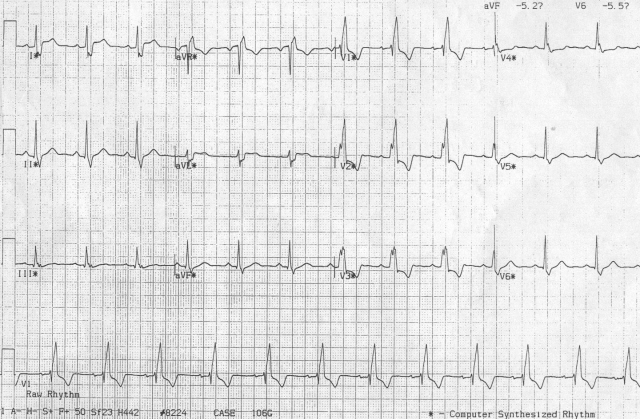
Baseline ECG with wide QRS (RBBB)

**Figure 1b:**
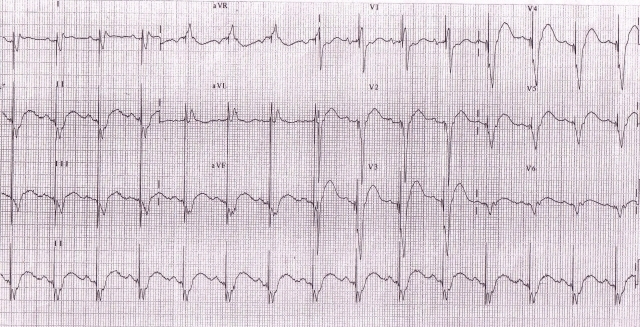
Post implant ECG

**Figure 2a:**
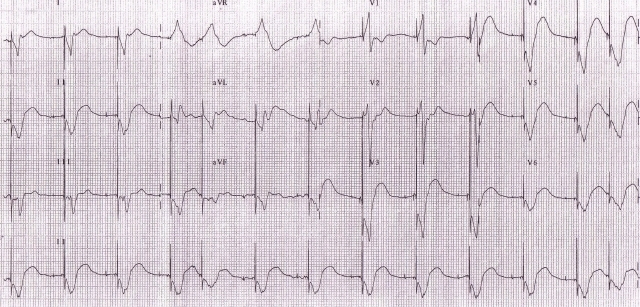
ECG showing widening of paced QRS due to hyperkalemia

**Figure 2b:**
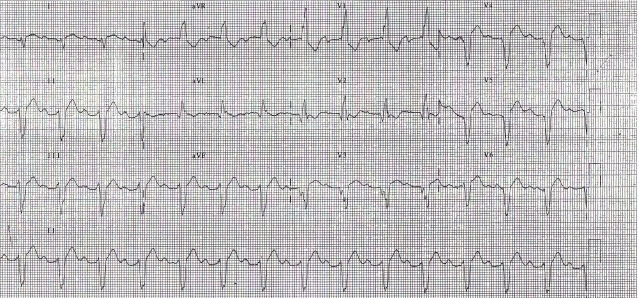
ECG with device in switched off mode

**Figure 3a:**
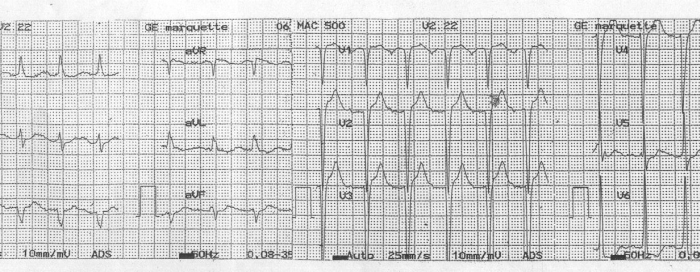
Baseline ECG with wide QRS (lBBB)

**Figure 3b:**
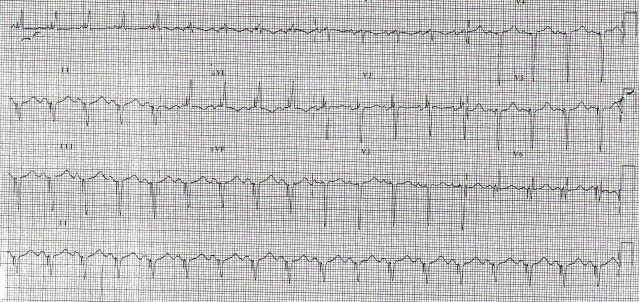
Post implant ECG

**Figure 4a:**
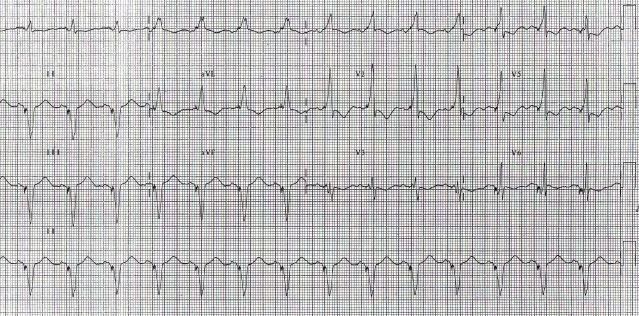
ECG showing widening of paced QRS due to progressive disease

**Figure 4b:**
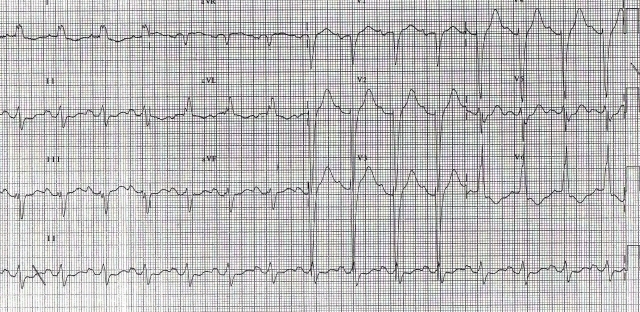
ECG with device in switched off mode
